# A Condemnation of Specialism in Medicine

**Published:** 1884-12

**Authors:** 


### A CONDEMNATION OF SPECIALISM IN MEDICINE.

In his work on *Sexual Neurasthenia*, Dr. Beard wrote this condemnation of the treatment of "local" disease as now practiced by specialists:

"The body of the sensitive man is a microcosm of reflex actions, and the three great centres of reflex irritation—the family of reflex centres—are the brain, the stomach, the genital system; between these messengers of evil or of good are ever passing in sleeping and in waking hours. To touch one is to touch all. These three are literally a trinity—three in one, one in three; they cannot be isolated.

"Besides these three general centres, there are sub-centres, all of importance, all to be considered in the study of nervous diseases—the spine, the eyes, the teeth, the glans penis, the ovaries—for disease of any of these parts may cause disease of any other part.

"From this general and demonstrable and important fact, false reasoning unlimited has sought to show that *all* functional nervous diseases whatsoever come from the eye, and that right glasses are a specific for neuroses; that the removal of the ovaries is the true treatment of neurasthenic women; that all nervousness, including morbid fears and morbid impulses, must depart after surgery has cured a lacerated cervix; that the opening of a stricture opens the door of escape for every other disease that afflicts the sufferer. Disappointments increasing and beyond enumeration attend those who look only at *one* of these many centres of reflex irritation and see not the others."

